# Engineering sorghum for higher 4-hydroxybenzoic acid content

**DOI:** 10.1016/j.mec.2022.e00207

**Published:** 2022-09-21

**Authors:** Chien-Yuan Lin, Yang Tian, Kimberly Nelson-Vasilchik, Joel Hague, Ramu Kakumanu, Mi Yeon Lee, Venkataramana R. Pidatala, Jessica Trinh, Christopher M. De Ben, Jutta Dalton, Trent R. Northen, Edward E.K. Baidoo, Blake A. Simmons, John M. Gladden, Corinne D. Scown, Daniel H. Putnam, Albert P. Kausch, Henrik V. Scheller, Aymerick Eudes

**Affiliations:** a Joint BioEnergy Institute, Emeryville, CA, 94608, USA; b Environmental Genomics and Systems Biology Division, Lawrence Berkeley National Laboratory, Berkeley, CA, 94720, USA; c Department of Cell and Molecular Biology, University of Rhode Island, Rhode Island, RI, 02892, USA; d Biological Systems and Engineering Division, Lawrence Berkeley National Laboratory, Berkeley, CA, 94720, USA; e Department of Plant Sciences, University of California-Davis, Davis, CA, 95616, USA; f Department of Biomaterials and Biomanufacturing, Sandia National Laboratories, Livermore, CA, 94551, USA; g Energy & Biosciences Institute, University of California-Berkeley, Berkeley, CA, 94720, USA; h Energy Analysis and Environmental Impacts Division, Lawrence Berkeley National Laboratory, Berkeley, CA, 94720, USA; i Department of Plant and Microbial Biology, University of California, Berkeley, CA, 94720, USA

**Keywords:** Sorghum, Bioproduct, 4-Hydroxybenzoic acid, Shikimate, Bioenergy crop, 4-HBA, 4-hydroxybenzoic acid, CaMV, cauliflower mosaic virus, CWR, cell wall residue, DAHP, 3-deoxy-D-arabino-heptulosonate, HPLC-ESI-TOF-MS, high performance liquid chromatography electrospray ionization and time-of-flight mass spectrometry, RT-qPCR, reverse transcription quantitative PCR, RuBisCo, ribulose-1,5- bisphosphate carboxylase

## Abstract

Engineering bioenergy crops to accumulate coproducts *in planta* can increase the value of lignocellulosic biomass and enable a sustainable bioeconomy. In this study, we engineered sorghum with a bacterial gene encoding a chorismate pyruvate-lyase (*ubiC*) to reroute the plastidial pool of chorismate from the shikimate pathway into the valuable compound 4-hydroxybenzoic acid (4-HBA). A gene encoding a feedback-resistant version of 3-deoxy-d-arabino-heptulonate-7-phosphate synthase (*aroG*) was also introduced in an attempt to increase the carbon flux through the shikimate pathway. At the full maturity and senesced stage, two independent lines that co-express *ubiC* and *aroG* produced 1.5 and 1.7 dw% of 4-HBA in biomass, which represents 36- and 40-fold increases compared to the titer measured in wildtype. The two transgenic lines showed no obvious phenotypes, growth defects, nor alteration of cell wall polysaccharide content when cultivated under controlled conditions. In the field, when harvested before grain maturity, transgenic lines contained 0.8 and 1.2 dw% of 4-HBA, which represent economically relevant titers based on recent technoeconomic analysis. Only a slight reduction (11–15%) in biomass yield was observed in transgenics grown under natural environment. This work provides the first metabolic engineering steps toward 4-HBA overproduction in the bioenergy crop sorghum to improve the economics of biorefineries by accumulating a value-added coproduct that can be recovered from biomass and provide an additional revenue stream.

## Highlights

• First report of 4-hydroxybenzoic acid overproduction in sorghum.
• Up to 40-fold increase in 4-hydroxybenzoic acid titers in biomass (1.7 dw%).
• Field-grown engineered sorghum accumulates 4-hydroxybenzoic at relevant titers.

## 1 Introduction

Lignocellulosic biomass can be a sustainable source of sugars for the manufacturing of bioproducts such as biofuels. However, the high costs associated with crop cultivation and deconstruction of biomass to simple sugars pose some challenges to the commercialization of advanced bioproducts (Baral et al., 2019). One proposed solution to improve the economics of biofuels is the *in-planta* accumulation of value-added coproducts (Yang et al., 2020). In this scenario, engineered bioenergy crops not only provide carbohydrates for conversion into bioproducts but also produce valuable compounds such as polymers, platform chemicals, pharmaceuticals, flavors and fragrances that can be extracted from biomass, purified, and sold (Lin and Eudes, 2020).

Sorghum is an ideal bioenergy feedstock due to its high water use efficiency, high biomass yields, and efficient nitrogen recycling (Mullet et al., 2014). Although several studies reported on the genetic modification of sorghum to reduce its recalcitrance to deconstruction, there are only a few examples of metabolic engineering for accumulation of bioproducts in sorghum biomass (Kempinski et al., 2019; Vanhercke et al., 2019). In this work, we tested the feasibility of engineering sorghum to overproduce 4-hydroxybenzoic acid (4-HBA). 4-HBA can serve as precursor for the manufacturing of nutraceuticals (e.g. coenzyme Q10, gastrodin, and resveratrol), cosmetic ingredients (e.g. arbutin), drugs (e.g. xiamenmycin and shikonin), fibers (e.g. Vectran™), platform chemicals (e.g. muconate), and deep eutectic solvents (Wang et al., 2018, 2021). Our recent technoeconomic analysis indicated that accumulating 4-HBA at sufficient titers in sorghum biomass has the potential to improve the economics of biorefineries and offers a competitive advantage compared to microbial synthesis routes (Yang et al., 2022). This analysis considered a process involving a preliminary extraction of 4-HBA from sorghum biomass while the remaining lignocellulose is further deconstructed and converted into ethanol by fermentation. The results showed that achieving a minimum 4-HBA titer of 0.3 dw% in biomass would be sufficient to reach cost-parity with microbial production of 4-HBA from glucose, even if microbial platforms were to reach theoretical maximum yields (Yang et al., 2022).

**Fig. 1:**
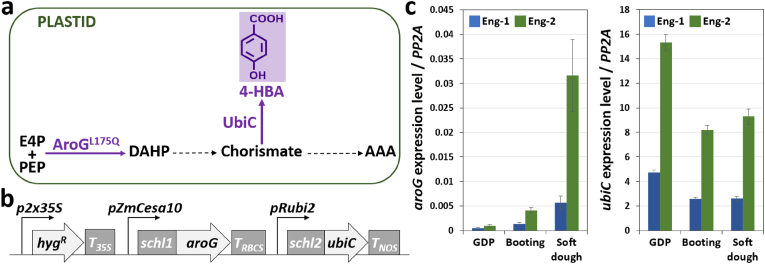
Engineering strategy for 4-HBA overproduction in sorghum. (a) Diagram of the shikimate pathway and engineering approach. The two *E. coli* enzymes targeted to plastids are 3-deoxy-D-arabino-heptulosonate (DAHP) synthase with L175Q mutation (AroG^L175Q^) and chorismate pyruvate-lyase (UbiC). Abbreviations: AAA: aromatic amino acids; E4P, erythrose 4-phosphate; PEP, phosphoenolpyruvate. (b) DNA construct used for sorghum transformation. *Schl1* and *schl2* encode transit peptides from pea and maize ribulose-1,5-bisphosphate carboxylase (RuBisCo) small subunits, respectively. *pZmCesa10* and *pRubi2* designate the promoters of maize cellulose synthase10 and rice polyubiquitin2 genes. *p2x35S* is the enhanced 35S promoter from cauliflower mosaic virus (CaMV). *T_35S_*, *T_RBCS_*, and *T_NOS_* are the terminators from CaMV 35S, Arabidopsis RuBisCo small subunit, and *Agrobacterium* nopaline synthase genes, respectively. *HygR* denotes the aminoglycoside phosphotransferase marker gene used for plant selection. See also Supplementary Table S1. (c) Transgene expression in two independent engineered lines (Eng-1 and Eng-2). *AroG* (left panel) and *ubiC* (right panel) transcripts were detected by RT-qPCR using mRNA obtained from the bottom part of the main stem at three different developmental stages. Transcript abundance relative to that of the *PP2A* sorghum gene is shown. Wild-type segregants were used as negative controls. Values are means ± SE of four biological replicates (n = 4 plants). GDP: growth differentiation point stage.

One engineering approach for *in-planta* overproduction of 4-HBA consists in the expression of *Escherichia coli* chorismate pyruvate-lyase (UbiC) targeted to plastids to reroute chorismate away from the shikimate pathway. For example, tobacco and sugarcane transformed with *ubiC* contained up to 0.52 and 0.69 dw% of 4-HBA in leaves, respectively, which were accumulated mostly as 4-HBA phenolic glucoside (McQualter et al., 2005; Siebert et al., 1996). Moreover, expression of plastid-targeted feedback-resistant 3-deoxy-d-arabino-heptulonate-7-phosphate synthase (DAHPS, AroG^L175Q^) from *E. coli* was shown to enhance the synthesis of metabolites that derive from the shikimate pathway in several plants (Eudes et al., 2018; Lin et al., 2021; Oliva et al., 2020; Tzin et al., 2012, 2013). In this study, we transformed sorghum with a construct for dual expression of *ubiC* and *aroG*^*L175Q*^ in order to boost 4-HBA content in biomass (Fig. 1a).

## 2 Materials and methods

### 2.1 Plant growth conditions and sampling

Plants were grown at the UC Berkeley South Greenhouse Oxford facility with a minimum temperature set at 22 °C. Seeds were germinated directly on soil (Sunshine mix #4, Sun Gro, Agawam, MA) in one-gallon pots. One tablespoon of Osmocote Plus 15-9-12 was added to the soil biweekly until the flowering stage. T1 and T2 plants were grown until seeds reached the black layer stage (i.e. full physiological maturity), after which watering was stopped and pots were allowed to dry for another 3 weeks. Growth parameters were measured at physiological maturity. For cell wall and 4-HBA analyses, plants were harvested without their panicles, further dried in an oven at 50 °C for five days, and ground into powder using a Mixer Mill MM 400 (Retsch Inc., Newtown, PA) and stainless-steel balls.

### 2.2 Design of the pZmCesa10:aroG-pRubi2:ubiC construct

The *pZmCesa10:aroG-pRubi2:ubiC* construct was obtained using the jStack cloning method (Shih et al., 2016) and the level-0 and level-1 intermediate plasmids are listed in Supplementary Table S1. For level-0 parts, the promoter sequence of the maize (*Zea mays*) cellulose synthase10 gene and the coding sequences of feedback-resistant DAHPS (AroG^L175Q^) and of chorismate pyruvate-lyase (UbiC) from *E. coli,* both preceded with a sequence encoding a plastid transit peptide and codon-optimized for expression in sorghum, were synthesized by GenScript (Piscataway, NJ). Sequences were flanked with BsaI or BsmBI restriction sites for level-1 cloning. The *pRubi2* promoter from rice (*Oryza sativa*) was amplified by PCR with primers containing BsaI restriction sites (Supplementary Table S2) using a plasmid as template (a gift from Roger Thilmony, USDA) and subcloned into the backbone pBca9145 by In-Fusion cloning (Takara Bio USA, Mountain View, CA). Plasmid sequences are available at the Inventory of Composable Elements (ICE) source registry (http://public-registry.jbei.org).

### 2.3 Sorghum transformation and genotyping

The *Agrobacterium tumefaciens* strain AGL1 was used to transform the grain sorghum variety BTx430 (*Sorghum bicolor* (L.) Moench) as previously described, except that hygromycin (20 μg mL^−1^) was used for plant selection (Do et al., 2018). In the T1 generation, TaqMan Real-time PCR assays were performed on gDNA using primers specific to the octopine synthase terminator (*tOCS*) from the T-DNA and to the *Actin7* reference gene to identify homozygous plants for the transgene and wild-type segregants (ARQ Genetics, Bastrop, TX). T1 and T2 plants were grown to full maturity to generate viable seeds.

### 2.4 Reverse transcription-quantitative PCR (RT-qPCR)

Total RNAs were extracted from the 5-cm bottom part of the main stem from plants in the T2 generation at three different developmental stages using the RNeasy Plant Mini Kit (Qiagen, Redwood City, CA). cDNA synthesis was conducted using the SuperScript IV First-Strand Synthesis kit (Thermo Fisher Scientific, Waltham, MA) as previously described (Hao et al., 2021). RT-qPCR was performed using 35 cycles consisting of 5 s at 95 °C for denaturation and 15 s at 60 °C for annealing and amplification. The relative quantification of *aroG* and *ubiC* transcripts was calculated using the 2-^Δ^CT method and normalized to the reference gene *PP2A* (NCBI Reference Sequence: XM_002453490.2) as previously described (Tian et al., 2021). The results are the average from four biological replicates. RT-qPCR primers are listed in Supplementary Table S2.

### 2.5 Metabolite extraction and quantification

Metabolites were extracted from 20 mg of dry biomass powder using 80% (v/v) methanol-water as solvent as previously described (Eudes et al., 2012). Free 4-HBA and 4-HBA glucose conjugates were analyzed using high-performance liquid chromatography-electrospray ionization time-of-flight-mass spectrometry (HPLC-ESI TOF-MS) (Eudes et al., 2013). Quantification was performed via 6-point calibration curves of standard compounds.

### 2.6 Cell wall composition analysis

Ball-milled biomass (1 g) was sequentially extracted using a Dionex ASE 350 accelerated solvent extractor set to 7-min static extraction cycles (Thermo Fisher Scientific, Waltham, MA). Solvents (5 ml) were water (two cycles), 80% (v/v) ethanol:water (ten cycles), 50% (v/v) methanol:chloroform (one cycle), and acetone (one cycle). Klason lignin and cell wall monosaccharides were determined on cell wall residues (CWR) using the standard NREL biomass procedure (Sluiter et al., 2008). Glucose, xylose, and arabinose from biomass hydrolysates were measured by HPLC as previously described (Lin et al., 2021). Cell-wall-bound aromatics were released from 20 mg of CWR via mild alkaline hydrolysis using a 2N sodium hydroxide solution (Eudes et al., 2012). Ferulate, *p*-coumarate, and 4-HBA were quantified using HPLC-ESI-QTOF-MS analysis (Eudes et al., 2013).

### 2.7 Saccharification assays

Biomass samples (200 mg) were pretreated with the ionic liquid cholinium phosphate for 3 h at 121 °C and the enzymatic saccharification was conducted at 50 °C for 72 h using an enzyme mixture of Cellic® CTec3 and HTec3 (9:1 v/v) (Novozymes, Bagsværd, Denmark) as previously described (Tian et al., 2021). Conditions used for saccharification assays (ionic liquid concentration, pretreatment temperature, and enzyme loadings) are considered to be suboptimal for total polysaccharide hydrolysis. For metabolite measurements, hydrolysates were filtered using 0.2 μM PVDF filters (EMD Millipore, Billerica, MA, USA) and analyzed by HPLC (Tian et al., 2021).

### 2.8 Field trial

Seeds (T3 generation) from lines Eng-1, Eng-2, pooled WT-1 and WT-2 (i.e., ‘wild-type segregants’), and untransformed wildtype (variety BTx430) were planted in a field trial conducted at the University of California Davis Plant Sciences Research Farm in 2021 under a USDA-APHIS Biotechnology Regulatory Services permit for regulated sorghum (#BRS 20-356-102r). Seeds were planted on a Yolo clay loam soil (fine-silty, mixed thermic Fluventic Haploxerept) on May 28, 2021. Percent germination was taken for each line, and adjusted for a target seeding rate of 197,600 seeds ha^−1^. The experiment was a randomized complete block design with four replicates. Row spacing was 0.76 m. Plots measured 3 × 6 m and comprised 4 rows. A commercial sorghum variety (NK8828 from S&W Seed Company, Longmont, CO) was also planted as border rows to limit edge effects. Total N-P-K fertilizer was applied at planting at a rate of 224, 91, and 22 kg ha^−1^, respectively. Sivanto was applied on June 22, 2021 at a rate of 481 g ha^−1^ for sugarcane aphid control. Irrigation water was applied utilizing surface furrow methods to satisfy the fully-watered evapotranspiration requirement for sorghum during the growing season. All panicles on each plant were covered with pollinating bags prior to anthesis to prevent pollen flow. Plot harvest took place on September 21, 2021. Panicles (heads with immature grain) were removed by hand. Remaining stover was harvested using a Wintersteiger Cibus forage chopper (Wintersteiger, Salt Lake City, UT). Subsamples of approximately 500 g of fresh weight stover material were taken from each harvested plot, and dried at 55 °C for one week in a forced air oven to determine dry matter and calculate yield. For 4-HBA analyses, stover samples were first milled using a Model 4 Wiley Mill equipped with a 1-mm mesh (Thomas Scientific, Swedesboro, NJ) and further ground into powder.

## 3 Results and discussion

### 3.1 Generation of aroG-ubiC transgenic sorghum

The sorghum variety BTx430 was transformed with a construct containing *ubiC* as well as the gene encoding a feedback-resistant version of DAHPS (AroG^L175Q^) from *E. coli* (Fig. 1b). In this construct, both *E. coli* genes were codon-optimized for expression in sorghum and preceded with the sequence of a signal peptide for targeting UbiC and AroG^L175Q^ to plastids (Fig. 1b). The promoter of the rice polyubiquitin gene (*pRubi2*) was used to drive *ubiC* expression constitutively whereas the promoter of a maize cellulose synthase gene (*pZmCesa10*) involved in the formation of secondary cell walls was selected for *AroG*^*L175Q*^ expression to avoid possible toxicity and sterility issues (Oliva et al., 2020) (Fig. 1b). Two independent lines (Eng-1 and Eng-2) containing the dual *aroG*-*ubiC* construct and their respective wild-type segregants (WT-1 and WT-2) were selected for further characterization. Using RT-qPCR, the expression of the two transgenes was confirmed in both lines at three different developmental stages in the T2 generation. In the bottom part of the main stem, *ubiC* expression level was higher than that of *aroG*, and line Eng-2 exhibited higher abundance of both *ubiC* and *aroG* transcripts compared to Eng-1 (Fig. 1c). The transgenics showed normal growth and were phenotypically indistinguishable from wild-type controls (Supplementary Fig. S1). Overall, no differences in various parameters such as the number of days to panicle emergence, height of the main tiller, stover biomass yield, seed weight, and estimated seed number were observed between transgenics and control plants grown under controlled environment (Supplementary Fig. S1).

### 3.2 Content of 4-hydroxybenzoic acid in engineered sorghum

**Fig. 2:**
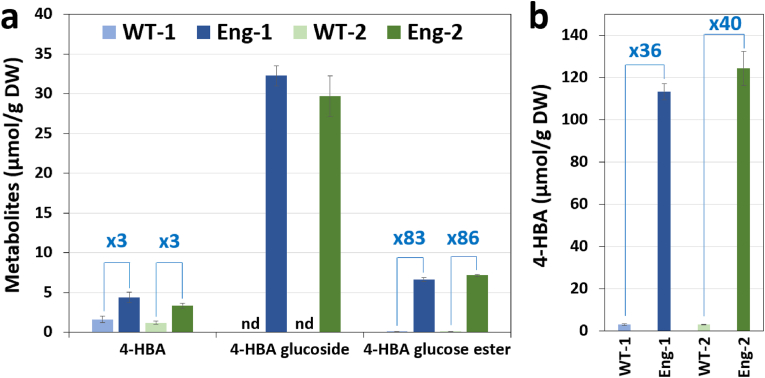
Metabolite analysis of engineered sorghum. (a) Titers of 4-HBA and its glucose conjugates extracted from of Eng-1 and Eng-2 using aqueous methanol. nd, not detected. (b) Total extractable 4-HBA content in Eng-1 and Eng-2 after acid-hydrolysis of the methanolic extracts. The stover from fully mature senesced dry plants in the T2 generation was analyzed. Values are means ± SE of five biological replicates (n = 5 plants). DW, dry weight.

Fully mature senesced dry plants in the T2 generation were ground into powder for metabolite analyses. Using liquid chromatography-mass spectrometry, measurements of free 4-HBA and potential 4-HBA glucose conjugates were analyzed in methanolic extracts obtained from biomass samples. A small amount of free 4-HBA was detected in wild-type extracts (1.2–1.6 μmol/g dw) and increased 3-fold in engineered lines (Fig. 2a). 4-HBA phenolic glucoside, which was undetectable in wild-type extracts, reached 32.3 and 29.7 μmol/g dw in extracts from lines Eng-1 and Eng-2, respectively (Fig. 2a). Furthermore, 4-HBA glucose ester was detected and increased 83- and 86-fold in transgenics, which corresponds to titers of 6.6 and 7.1 μmol/g dw (Fig. 2a). An aliquot of the methanolic extracts was subjected to acid hydrolysis to release 4-HBA from its conjugated forms. Measurement of 4-HBA aglycone in resulting hydrolysates showed that transgenic lines Eng-1 and Eng-2 contain 113 and 124 μmol 4-HBA/g dw, which is equivalent to 4-HBA titers of 1.56 and 1.72 dw% and represents 36- and 40-fold increases compared to the titer measured in wildtypes (Fig. 2b). Since the total amount of free 4-HBA and 4-HBA glucose conjugates in methanolic extracts is below that of 4-HBA aglycone measured in acid-treated extracts, we conclude that other unidentified 4-HBA conjugate forms accumulate in transgenics.

**Fig. 3:**
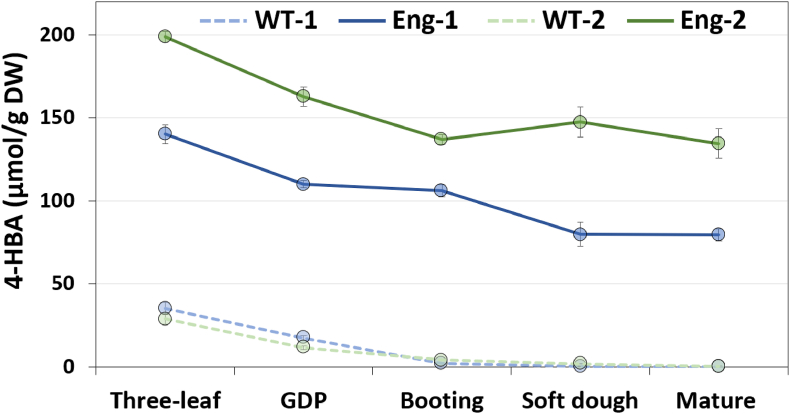
4-HBA analysis in engineered sorghum across different developmental stages. Titers of 4-HBA after acid-hydrolysis of methanolic extracts are shown. The stover from oven-dried plants in the T3 generation was analyzed. Values are means ± SE of five biological replicates (n = 5 plants). Abbreviation: DW, dry weight; GDP, growth differentiation point stage.

New cultures were initiated to assess 4-HBA titers during plant development in the T3 generation: Across five developmental stages, the data show that 4-HBA content measured in acid-treated methanolic extracts tends to decrease with plant age until flowering (‘booting’) and that line Eng-2 accumulates more 4-HBA than line Eng-1 at all stages (Fig. 3). The measurement of higher amount of 4-HBA in line Eng-2 compared to Eng-1 is consistent with the expression level of the transgenes in these lines, however, this trend was not observed in the T2 generation in which Eng-1 and Eng-2 plants produced comparable amounts of 4-HBA (Fig. 2b). Since all the plants analyzed were homozygous for the DNA construct, and considering that the two transgenic lines should be genetically stable in the T2 and T3 generations, differences in growing conditions that may have occurred in the greenhouse during the cultivation of T2 and T3 plants could explain these observations.

### 3.3 Cell wall analyses in engineered sorghum

Cell walls are the main constituents of plant biomass and the source of fermentable substrates for the production of advanced bioproducts. The analysis of cell wall composition showed no significant difference between transgenics and controls regarding the proportion of major components including glucose (representing cellulose), xylose and arabinose (representing xylan), and lignin, as well as the aromatics *p*-coumarate, and ferulate (Supplementary Table S3). However, 4-HBA content measured in alkaline hydrolysates obtained from extractive-free cell wall preparations was six and four times higher in transgenics compared to controls (Supplementary Table S3). In a separate study that investigated the lignin monomeric composition in line Eng-2, we evidenced that 4-HBA formed ester end-groups on lignin (Wang et al., 2021), which was previously described in lignin fractions isolated from other plants such as willow, poplar, aspen, Neptune grass, and palms (del Río et al., 2020).

The saccharification of sorghum biomass was evaluated using ionic liquid pretreatment followed by enzymatic hydrolysis using a mixture of cell wall degrading enzymes that convert cellulose and hemicellulose into simple sugars. The biomass from engineered lines released similar amount of glucose compared to wild-type controls, but xylose yields were reduced by 6.7% and 8.5% in Eng-1 and Eng-2, respectively (Supplementary Fig. 2). Overall, this data indicates that cell walls from engineered sorghum are not substantially more recalcitrant to enzymatic hydrolysis, which is an important consideration since degrading enzymes represent an important cost in lignocellulosic biorefineries (Klein-Marcuschamer et al., 2012). Ferulate has been implicated in the formation of phenolic cross-links that reinforce the cell wall, but the involvement of 4-HBA in such a role has not been described (Mnich et al., 2020). It is plausible that 4-HBA accumulated in the cell wall of Eng-1 and Eng-2 participates in the formation of cross-links that somehow impact slightly the hydrolysis of xylan chains during saccharification.

### 3.4 Field-testing of engineered sorghum

**Fig. 4:**
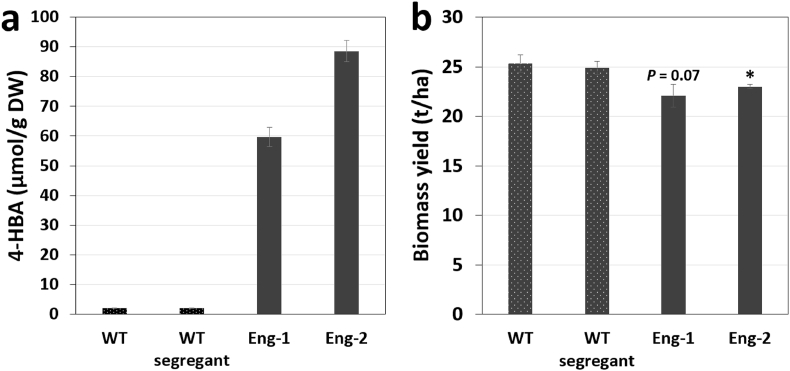
Field testing of engineered sorghum. (a) 4-HBA titers and (b) stover biomass yields are shown. The stover from plants in the T3 generation grown until the soft dough stage was analyzed. Values are means ± SE of four biological replicates (n = 4 plots). ‘WT’ is conventional sorghum (variety BTx430) and ‘WT segregant’ is a pool of WT-1 and WT-2 segregants. The asterisk indicates a significant difference compared to the wildtypes using the unpaired Student's t-test (*P < 0.05). DW, dry weight.

The engineered sorghum lines were grown in the field in the T3 generation to evaluate their performance under natural environment. For this field trial, an equal amount of seeds from each wild-type segregants was pooled to generate a single wild-type segregant control. Seeds from a wildtype of the same variety that did not go through the transformation process was included as a second control. Plants were grown until the soft dough stage and methanol-soluble metabolites were extracted from the stover. As previously observed in the greenhouse experiment, the two engineered sorghum lines showed a large increase of 4-HBA content (30 and 44-fold) compared to control plants, which averaged 59.7 and 88.8 μmol/g DW in Eng-1 and Eng-2, respectively (Fig. 4). These titers, which are equivalent to 8.1 and 12.0 mg/g DW (or 0.8 and 1.2 dw%), are significantly lower than those measured in plants analyzed at the same developmental stage and grown in the greenhouse, but the exact reasons for this difference remain to be elucidated. Measurement of stover biomass showed slight decreases in yield for both transgenic lines (15% and 11%) compared to the wild-type controls that produced 24.9–25.3 tonnes per hectare (Fig. 4).

## 4 Conclusions

This work demonstrates that sorghum can be engineered to accumulate the valuable bioproduct 4-HBA at potentially economically competitive titers compared to microbial synthesis (i.e. > 0.3 dw%; Yang et al., 2022). Measurements of 4-HBA content during sorghum development suggests that engineered plants could be harvested either at the soft dough stage or at full maturity since 4-HBA titers remain comparable between the two stages after flowering (Fig. 3). Although higher biomass yields are typically obtained at the mature stage, the soft dough stage represents the harvest time for silage and several studies have highlighted the economic potential of using forage sorghum for biorefinery applications (Magurudeniya et al., 2021; Yang et al., 2021). Line Eng-2, which showed higher 4-HBA titers compared to Eng-1, could be further engineered to enhance 4-HBA content. Possible strategies include the additional expression of UbiC targeted to the cytosol where its substrate chorismate is also located (Qian et al., 2019; Sommer and Heide, 1998), and the expression of the recently discovered *p*-hydroxybenzoyl-CoA monolignol transferase to promote the attachment of readily cleavable 4-HBA esters onto lignin in cell walls (de Vries et al., 2022; Zhao et al., 2021). Considering the slight yield penalty observed in line Eng-2 under field conditions, the 4-HBA co-product trait developed in this line should be introgressed into high-yielding biomass sorghum varieties (Olson et al., 2012). Further field testing of 4-HBA-rich sorghum is needed to evaluate crop performance under various conditions such as reduced irrigation since bioenergy crops are expected to be grown on marginal lands that do not compete with food production.

## Funding

This work conducted by the Joint BioEnergy Institute was supported by the US 10.13039/100000015Department of Energy, 10.13039/100006132Office of Science, Office of 10.13039/100006206Biological and Environmental Research under contract no. DE-AC02-05CH11231 between Lawrence Berkeley National Laboratory and the US Department of Energy. Part of the work was supported by the 10.13039/100007000Laboratory Directed Research and Development Program of 10.13039/100006235Lawrence Berkeley National Laboratory (J.T. and T.R.N).

## Author statement

C-YL and JT performed DNA clonings. APK, JH, and KN-V transformed sorghum. MYL and YT genotyped the plants. C-YL conducted gene expression analyses. YT measured agronomic traits and analyzed metabolites and cell wall components. VRP conducted saccharification assays. RK and EEKB performed LC-MS analyses. C.M.DB conducted the field trial and biomass handling. J.D. provided logistics for the field trial. AE wrote the paper. APK, TRN, EEKB, DHP, JMG, BAS, CDS, HVS, and AE supervised the research. All authors read and approved the final manuscript.

## Declaration of competing interest

The authors declare that they have no known competing financial interests or personal relationships that could have appeared to influence the work reported in this paper.
