# Supplementary material for: Engineering sorghum for higher 4-hydroxybenzoic acid content
Source: Metab Eng Commun. 2022 Sep 21;15:e00207. doi: 10.1016/j.mec.2022.e00207 (PMC9519784; doi:10.1016/j.mec.2022.e00207)
Supplement: Multimedia component 1 [file mmc1.pdf]

Engineering sorghum for higher 4-hydroxybenzoic acid content

Chien-Yuan Lin, Yang Tian, Kimberly Nelson-Vasilchik, Joel Hague, Ramu Kakumanu, Mi Yeon Lee, Venkataramana R. Pidatala, Jessica Trinh, Christopher M. De Ben, Jutta Dalton, Trent R. Northen, Edward E. K. Baidoo, Blake A. Simmons, John M. Gladden, Corinne D. Scown, Daniel H. Putnam, Albert P. Kausch, Henrik V. Scheller, Aymerick Eudes

Appendix A. Supporting information

Supplementary Table S1. List of plasmids used in this study.

Supplementary Table S2. Primers used in this study.

Supplementary Table S3. Chemical composition of cell walls obtained from biomass of sorghum engineered lines and wild-type segregants. Values in brackets are the SE from six biological replicates (*n* = 6).

Supplementary Figure S1. Agronomic parameters of lines Eng-1 and Eng-2 compared to wild-type controls in the T2 generation. Fully mature senesced plants were used for b-e. (**a**) Days to panicle emergence, (**b**) height of the main tiller, (**c**) stover biomass yield, (**d**) seed weight and (**e**) number of seeds per plant. (**f**) Image of the two independent engineered lines (Eng-1 and Eng-2) and respective wild-type segregant controls (WT-1 and WT-2) at physiological maturity and before senescence. Values are means ±SE of six biological replicates. No significant differences were observed between transgenics and wildtypes using the unpaired Student's t-test ($P < 0.05$). ns: No significant difference.

Supplementary Figure S2. Biomass saccharification of mature senesced sorghum lines in the T2 generation. Amounts of glucose and xylose released after ionic liquid pretreatment followed by 72-h of enzymatic hydrolysis with a cellulase and hemicellulose cocktail are shown. Values are means ±SE of six biological replicates (*n* = 6 plants). Asterisks indicate significant differences from wild-type controls using the unpaired Student's t-test (*$P < 0.05$).

Supplementary Table S1. List of plasmids used in this study.

Construct name	Level	Backbone	Description	JBEI ICE ID
pZmCesa10:aroG-pRubi2:ubiC	2	pPMS074	tOCS-Hyg^{R}-pZmCesa10:schl1-aroG^{L175Q}-tRbcS-pRubi2:schl2-ubiC-tNOS	JBx_101015
tOCS-Hyg^{R}-pZmCesa10:schl1-aroG^{L175Q}-tRbcS	1	pPMS028	Level-1 construct obtained with level-0 parts:<br>{L_tOCS-Hyg^{R}}<br>{P_ZmCesa10}<br>{C_schl1-aroG^{L175Q}}<br>{T_tRbcS}	JBx_100613
tRbcS-pRubi2:schl2-ubiC-tNOS	1	pPMS028	Level-1 construct obtained with level-0 parts:<br>{L_tRbcS}<br>{P_Rubi2}<br>{C_schl2-ubiC}<br>{T_tNOS}	JBx_092948
{L_tOCS-Hyg^{R}}	0	pBca9145	*Agrobacterium* octopine synthase terminator and plant hygromycin selectable marker, primary linker	JBx_065722
{P_pZmCesa10}	0	pBca9145	Promoter region of the maize cellulose synthase10 gene	JBx_092933
{C_schl1-aroG^{L175Q}}	0	pBca9145	Plastid-targeted feedback-resistant DAHPS from *E. coli* (WP_032246946)	JBx_092369
{T_tRbcS}	0	pBca9145	Arabidopsis Rubisco small subunit terminator	JBx_042282
{L_tRbcS}	0	pBca9145	Arabidopsis Rubisco small subunit linker	JBx_042288
{P_Rubi2}	0	pBca9145	Promoter region of the rice ubiquitin2 gene	JBx_092368
{C_schl2-ubiC}	0	pBca9145	Plastid-targeted chorismate pyruvate-lyase from *E. coli* (GenBank: EES9173034.1)	JBx_092545
{T_tNOS}	0	pBca9145	*Agrobacterium* nopaline synthase terminator	JBx_042266

Supplementary Table S2. Primers used in this study.

Primer name	Purpose / Target	Sequence (5'-3')
BsaI-pRubi2-Fw	Part isolation / *pRubi2*	cgctaaggatgatttctggaattcggtctcTggagATTCGGGTCAAGGCGGAAGCC
BsaI-pRubi2-Rv		cagctcgagttaggatccggtctcAcattGATCTGAATCTGCAAGAAATAATCAC
aroG-qPCR-Fw	RT-qPCR / *aroG* cDNA	GCGCACCGCATTGTTTCCT
aroG-qPCR-Rv		CAGTCCCCGTTTCCAGAGGT
ubiC-qPCR-Fw	RT-qPCR / *ubiC* cDNA	GATGGCCCCCACGGTTATGA
ubiC-qPCR-Rv		CGTTTCCAAGCGAACGCGAA

Supplementary Table S3. Chemical composition of cell walls obtained from biomass of sorghum engineered lines (Eng-1 and Eng-2) and wild-type segregants (WT-1 and WT-2). Values in brackets are the SE from six biological replicates (*n* = 6).

	**WT-1**	**Eng-1**	**WT-2**	**Eng-2**
Glucose (mg/g)	374.7 (1.2)	369.5 (3.9)	382.2 (5.1)	385.8 (2.4)
Xylose (mg/g)	210.9 (1.0)	211.6 (1.4)	212.8 (5.1)	213.0 (2.4)
Arabinose (mg/g)	26.7 (0.7)	27.9 (0.6)	25.6 (0.7)	25.4 (0.3)
Lignin (mg/g)	156.5 (2.6)	155.7 (3.2)	158.7 (3.8)	160.5 (1.4)
p-Coumarate (mg/g)	9.2 (1.2)	9.6 (0.5)	10.6 (0.1)	9.9 (0.8)
Ferulate (mg/g)	4.7(0.8)	4.6 (0.4)	5.1 (0.1)	4.6 (0.5)
4-HBA (μg/g)	31.7 (6.7)	**258.1 (19.8)***	50.3 (4.6)	**216.7 (28.8)***

Asterisks indicate a significant difference from the wildtype using the unpaired Student's t-test (*$P < 0.01$).

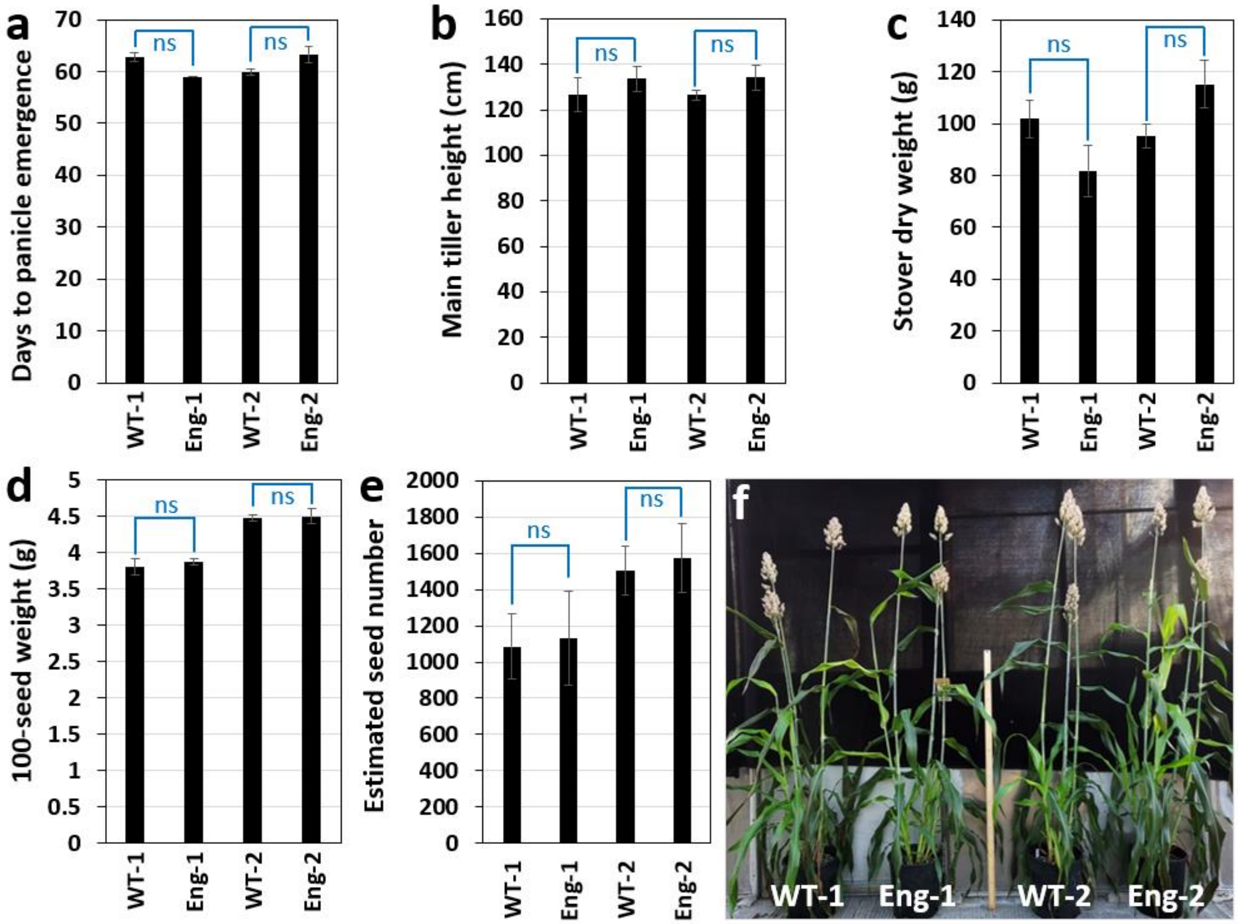

Supplementary Figure S1. Agronomic parameters of lines Eng-1 and Eng-2 compared to wild-type controls (WT-1 and WT-2) in the T2 generation. Fully mature senesced plants were used for b-e. (**a**) Days to panicle emergence, (**b**) height of the main tiller, (**c**) stover biomass yield, (**d**) seed weight and (**e**) number of seeds per plant. (**f**) Image of the two independent engineered lines and respective wild-type segregant controls at physiological maturity and before senescence. Values are means ±SE of six biological replicates. No significant differences were observed between transgenics and wildtypes using the unpaired Student's t-test ($P < 0.05$). ns: No significant difference.

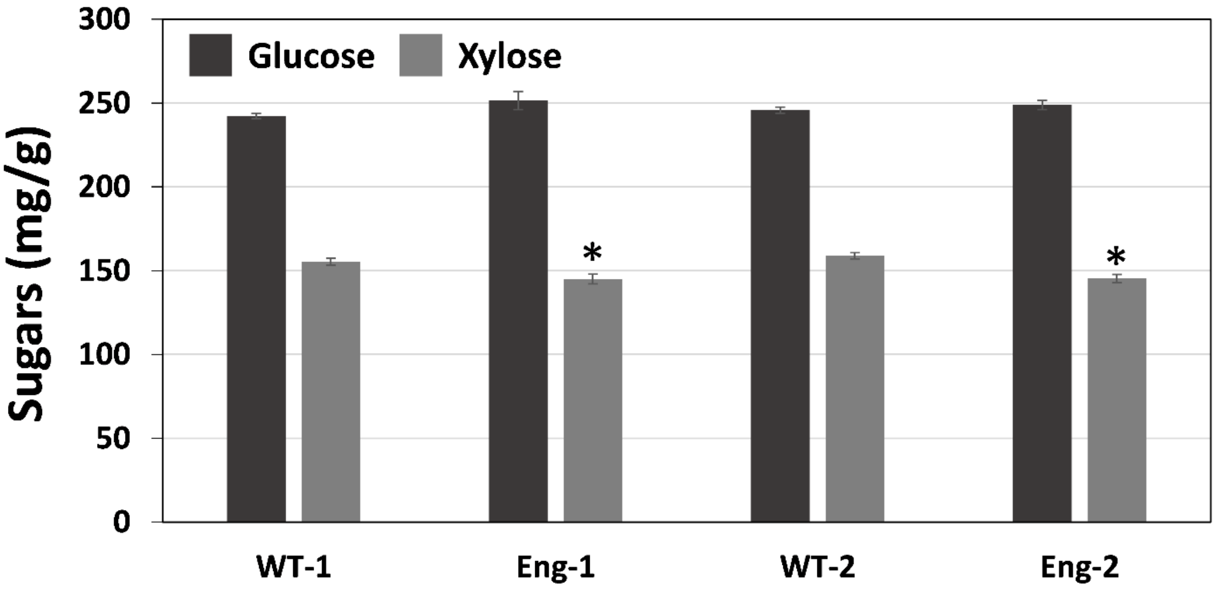

Supplementary Figure S2. Biomass saccharification of mature senesced sorghum lines in the T2 generation. Amounts of glucose and xylose released after ionic liquid pretreatment followed by 72-h of enzymatic hydrolysis with a cellulase and hemicellulose cocktail are shown. Values are means ±SE of six biological replicates (*n* = 6 plants). Asterisks indicate significant differences from wild-type controls using the unpaired Student's t-test (*$P < 0.05$).
